# Development and Evaluation of Filtration Performance of ZnO-Doped PEI Composite Membranes

**DOI:** 10.3390/polym18202499

**Published:** 2026-10-13

**Authors:** Güler Türkoğlu Demirkol

**Affiliations:** Department of Environmental Engineering, Faculty of Engineering, Istanbul University-Cerrahpasa, Avcılar, 34320 Istanbul, Turkey; gulertde@iuc.edu.tr

**Keywords:** polyetherimide, zinc oxide, nanocomposite membrane, phase inversion, ultrafiltration, antifouling

## Abstract

Polyetherimide (PEI)-based membranes were fabricated by phase inversion to examine the effects of zinc oxide (ZnO) nanoparticles and polyvinylpyrrolidone (PVP) on membrane structure, wettability, permeation, and fouling behavior. Six formulations comprising neat PEI, PEI/PVP, two ZnO loadings (0.5 and 1 wt.%), and the corresponding PVP/ZnO combinations were characterized by Fourier-transform infrared (FTIR) spectroscopy, scanning electron microscopy (SEM), casting-solution viscosity, contact angle, water content, gravimetric porosity estimation, effective pore-radius estimation, and sequential distilled-water/lake-water filtration. The additives produced marked but non-monotonic changes in solution viscosity, morphology, and surface wettability. PEI/PVP/ZnO-0.5 exhibited the lowest contact angle (approximately 35°), whereas PEI/PVP/ZnO-1 showed the smallest lake-water flux decline and the lowest total (approximately 4%) and irreversible (approximately 1%) fouling ratios. The results indicate that the effects of ZnO loading depend strongly on the presence of PVP and that wettability alone does not explain the observed fouling response. Under the present short-term filtration conditions, PEI/PVP/ZnO-1 provided the most favorable antifouling performance among the formulations tested.

## 1. Introduction

Polymeric membrane technologies have been widely used in water and wastewater treatment because of their high separation efficiency and low energy consumption. Polyetherimide (PEI) is a high-performance matrix polymer with high mechanical strength, remarkable thermal stability and chemical resistance, which is used widely in membrane fabrication. However, the hydrophobic property of PEI and its derivatives leads to fouling difficulties during the operation by adsorption of organic and inorganic particles onto the membrane surface. There has been a lot of interest in the creation of organic–inorganic nanocomposite membranes recently, where inorganic nanoparticles are introduced into the polymer matrix to decrease fouling and enhance membrane performance [1,2,3].

Zinc oxide (ZnO) nanoparticles are one of the most preferred additions for membrane modification because of their high hydrophilicity, chemical stability, low cost, and antifouling and photocatalytic characteristics [4,5]. When ZnO is introduced into the casting solution of membranes fabricated via the phase-inversion method, it directly affects the thermodynamics of phase separation (demixing) [4,5]. Studies in the literature have shown that ZnO nanoparticles increase the rate of solvent–non-solvent exchange in the coagulation bath, resulting in a thinner skin layer and wider macrovoids in the membrane structure [5,6]. Furthermore, the incorporation of ZnO markedly reduces the surface contact angle, confirming an increase in surface hydrophilicity [5,7].

Research on ZnO-doped polymeric nanocomposite membranes has demonstrated significant improvements in both permeability and fouling resistance [7,8]. ZnO nanoparticles facilitate the passage of water molecules through the membrane surface. The addition of ZnO at appropriate loadings (typically 0.5–2 wt.%) increases membrane porosity and achieves substantial gains in pure-water flux compared to pristine membranes [7,8]. However, excessive ZnO loading has been reported in numerous sources to cause particle agglomeration [8,9].

The thin hydration layer imparted to the membrane surface by ZnO physically prevents the adsorption of proteins (e.g., BSA), humic acids, and other organic foulants. Studies have shown that ZnO-containing membranes exhibit remarkably high flux recovery ratio (FRR) values and reduced irreversible fouling [8,10]. ZnO/polymer nanocomposite membranes have also demonstrated superior performance not only in fundamental filtration but also in the retention of specific pollutants. The active surface sites inherent in the ZnO structure enable high removal rates of synthetic dyes and heavy metal ions through adsorption and electrostatic repulsion mechanisms. Moreover, the well-known strong antibacterial nature of ZnO inhibits microbial growth and biofilm formation (biofouling) on the membrane surface, thereby extending the membrane service life and reducing operational costs [11,12].

In this study, nanocomposite ultrafiltration (UF) membranes were fabricated by incorporating ZnO nanoparticles into a hydrophobic PEI polymer via the phase-inversion method. PVP was also utilized as a pore-forming additive, and the combined effect of PVP and ZnO on the membrane structure was systematically investigated. ZnO thermodynamically modifies the membrane structure to maximize water flux, while its high antifouling and antibacterial properties enhance membrane stability. Accordingly, the developed PEI/ZnO nanocomposite membranes offer strong potential for the design of more sustainable, durable, and high-performance filtration systems in industrial water and wastewater treatment.

## 2. Materials and Methods

### 2.1. Materials

Polyetherimide (PEI, Mw = 30,000 g/mol) was procured from Sigma-Aldrich and used as the membrane matrix polymer. Zinc oxide (ZnO) nanoparticles, employed as the inorganic nanofiller, were also obtained from Sigma-Aldrich (St. Louis, MO, USA). Polyvinylpyrrolidone (PVP) was used as the pore-forming agent. N-Methyl-2-pyrrolidone (NMP) served as the solvent. Distilled water was used for the coagulation bath and all washing steps.

### 2.2. Membrane Fabrication

#### 2.2.1. Preparation of Neat PEI Membranes

Prior to the preparation of casting solutions, PEI was dried in an oven at 80 °C for 24 h to remove any residual moisture. The compositions of the membrane casting solutions are shown in Table 1.

First, 85 g of NMP was placed in 250 mL capped glass bottles. While the solution was being stirred on a heated magnetic stirrer (Velp ARE, Usmate, Italy) at 60 °C, 15 g of PEI was gradually added. The solution was left to stir for 24 h at 60 °C. After confirming a homogeneous appearance, the solution bottles were placed in an ultrasonic bath (Weightlab Instruments, İstanbul, Turkey) at room temperature (25 °C) for 30 min to remove entrapped air bubbles. The casting was performed using a glass plate and a casting knife (VF2170-261 TQC Sheen, Shanghai, China) set to a gap thickness of 200 µm. After spreading the solution on the glass plate, it was immersed in a coagulation bath containing distilled water, where the solvent (NMP) in the polymeric film exchanged with the non-solvent (water), inducing solidification of the polymeric film through phase inversion. The membranes separated from the plate and floated to the surface of the water bath. At least six membrane sheets were prepared from each casting solution. The fabricated membranes were thoroughly washed with deionized water to remove residual solvent and stored in distilled water at 4 °C until characterization.

#### 2.2.2. Fabrication of PEI/ZnO and PEI/PVP/nO Nanocomposite Membranes

Prior to the fabrication of the nanocomposite membrane, both PEI and ZnO were dried in an oven for 24 h at 80 °C. The PEI content was kept constant at 15 wt.% in all formulations, while ZnO was added at 0.5 and 1 wt.%. The glass bottles were charged with NMP, then the necessary amount of ZnO was added and dispersed at high stirring speed for 10 min at 60 °C, to achieve equal distribution in the solvent. Then PEI was added slowly and the casting solutions were agitated at 60 °C for 24 h. After homogeneity was confirmed, the solutions were ultrasonically mixed for 30 min at room temperature to eliminate air bubbles. The subsequent casting and phase-inversion method was identical to that of the plain PEI membranes described above. For the membranes containing PVP, PEI, followed by ZnO and finally PVP, was added to the solution in the order specified in the table, and the same procedure was continued.

### 2.3. Membrane Characterization

#### 2.3.1. Fourier-Transform Infrared Spectroscopy (FTIR)

The functional groups included in the prepared membranes were detected by utilizing FTIR spectrometer (Perkin Elmer Spectrum 100 (Springfield, IL, USA)). The spectra were acquired in the wavenumber range 4000–650 cm ^−1^.

#### 2.3.2. Electron Scanning Microscopy (SEM)

Scanning electron microscopy (SEM, FEI Quanta 250 FEG, Hillsboro, OR, USA) was used to study the morphologies of surfaces and cross-sections of the membranes at 5.00 kV under low-vacuum conditions (30 Pa). For sample preparation, membrane specimens were withdrawn from distilled water, and washed repeatedly with 30%, 60% and 100% ethanol/water solutions, then dried at room temperature. Cross-sectional samples were produced by fracturing the membrane in liquid N_2_. All the samples were sputter-coated with a thin layer of gold (Au) to make the surface electrically conductive before imaging.

#### 2.3.3. Measurements of Viscosity

The viscosities of the casting solutions were determined by viscometer (Fungilab Alpha, Barcelona, Spain) at 25 °C. Viscosity was calculated based on the average values obtained from three replicate measurements of the polymer solutions.

#### 2.3.4. Contact Angle Measurement

The contact angle goniometer (Attention Theta Flex, Gothenburg, Sweden) was used to evaluate the hydrophilicity of the manufactured membranes. A droplet of distilled water was placed on the membrane surface using a syringe and the contact angle between the water droplet and the membrane surface was measured. Measurements were repeated at three separate sites on each membrane sample and the average value was reported.

#### 2.3.5. Water Retention Capacity Measurement

The membranes were dried in an oven (NUVE EN 500, Ankara, Turkey) for 24 h at 60 °C and weighed in the dry condition. The dried membranes were immersed in distilled water. The excess surface water was rapidly wiped off with tissue paper and the wet bulk weighed. Three samples were selected from different sheets of each fabricated membrane, cut to the same area, and tested for water retention capacity in triplicate. The average values were then calculated. The capacity of water uptake was determined by Equation (1):

(1) Water content (%) = [(m_wet_ – m_dry_)/m_wet_] × 100

#### 2.3.6. Porosity

The membrane porosity (P) was obtained gravimetrically and computed from Equation (2):

(2) P (%) = [(m_wet_ – m_dry_)/(A × t × ρ)] × 100

Here, m_wet_ and m_dry_ are the wet and dry membrane masses (g), respectively; A is the membrane area (cm^2^); t is the membrane thickness (cm); and ρ is the density of water (0.998 g/cm^3^).

#### 2.3.7. Mean Pore Size

The mean pore radius (r_m_) of the membranes was calculated with the Guerout–Elford–Ferry equation (Equation (3)),

(3) $$r_{m}=\sqrt{\frac{\left(2.9-1.75Ɛ\right)8\eta \mathrm{lQ}}{ƐA∆P}}$$

where *Ɛ* is the membrane porosity, η is the viscosity of water (8.9 × 10^−4^ Pa·s), l is the membrane thickness (m), Q is the permeate volume per unit time (m^3^/s), A is the effective membrane area (m^2^), and ΔP is the transmembrane pressure (0.3 MPa).

#### 2.3.8. Thickness of Membrane

The thicknesses of the dried membranes were measured using a digital micrometer. Measurements were performed away from the border of the membranes to avoid distorted portions. Each membrane specimen was tested five times and the average value provided.

#### 2.3.9. Pure-Water Permeation Property

The membrane permeation performance was tested in a stirred dead-end filtration cell (Tin Engineering). The nitrogen gas was employed to drive water penetration. Membranes were cut to a defined area of 5 cm^2^ and placed in the filtration cell which was placed on a JKI magnetic stirrer. To reach a steady flux and to fully open the membrane pores, clean water was added to the 300 mL filtering cell and a compaction step was performed at 5 bar for 10 min. Then pure water was sent through at 3 bar. Permeate was collected in a beaker placed on an analytical balance and data was recorded digitally. Pure-water flux (J) was determined using Equation (4):

(4) $$J=\frac{V}{A\cdot ∆t}$$

where J is the flow (L/m^2^·h), V is the permeate volume (L), A is the membrane area (m^2^), and ∆t is the duration of time (h).

The membranes were first rinsed with distilled water, followed by three consecutive filtration cycles using lake water, and were subsequently rinsed again with distilled water. The initial water quality parameters of the lake water are presented in Table 2.

## 3. Results

### 3.1. FTIR Study

**Figure 1:**
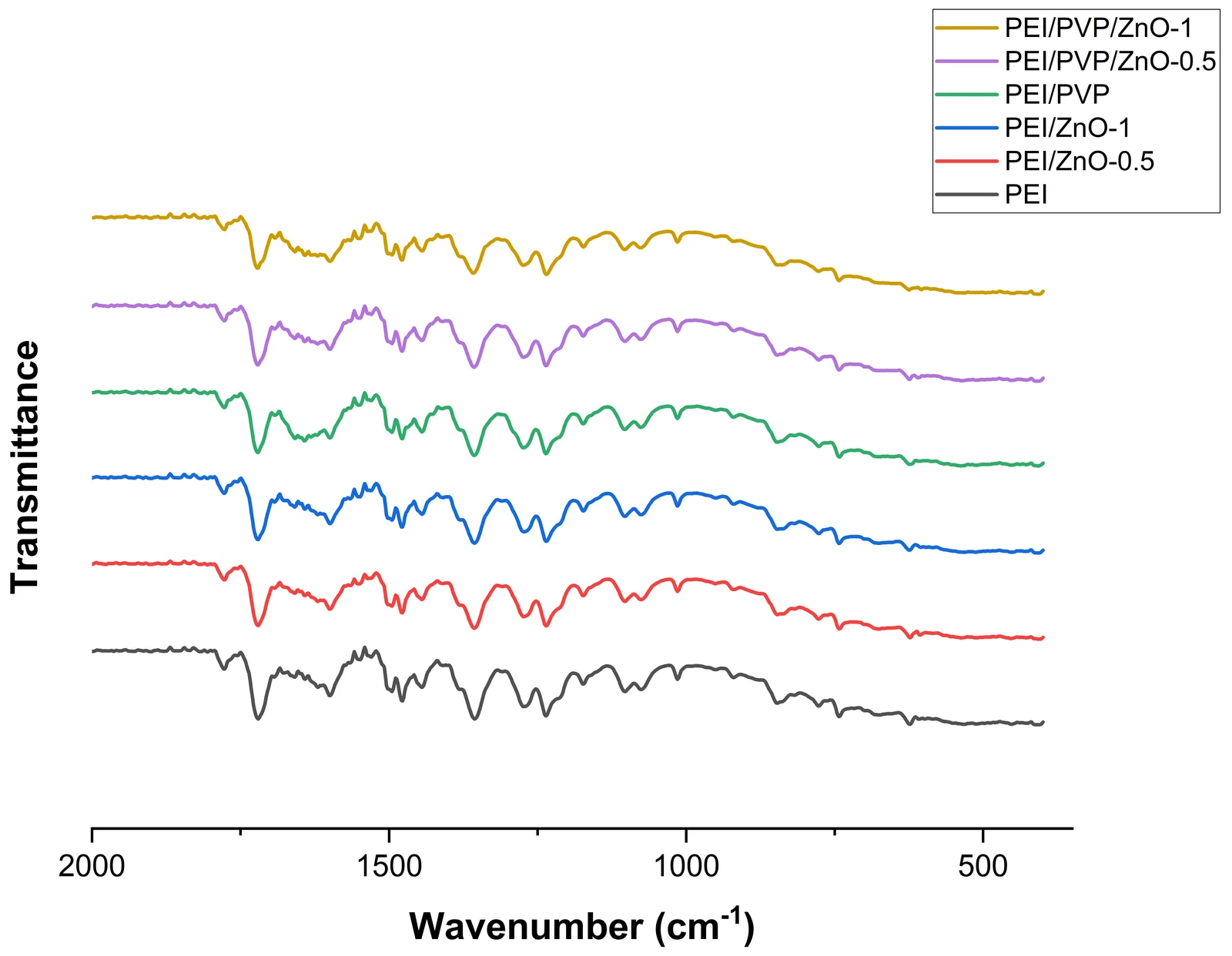
The FTIR spectra of the fabricated membranes.

Figure 1 presents the FTIR spectra of all fabricated membranes. The spectrum of the neat PEI membrane shows the characteristic absorption bands of polyetherimide. Two deep absorption peaks are seen at about 1780 and 1720 cm^−1^, which are attributed to asymmetric and symmetric carbonyl (C=O) stretching vibrations of the imide group, respectively. The absorption band at 1360 cm^−1^ is attributed to the C–N stretching vibration of the imide ring, and the peak near 740 cm^−1^ corresponds to the deformation of the imide ring [13,14,15]. The extra bands in the 1230–1270 cm^−1^ region are attributed to C–O–C asymmetric stretching of the aryl ether linkage and the band near 1500 cm^−1^ is attributed to aromatic C=C stretching vibrations [14,15].

The six membrane formulations, as shown in Figure 1, have the same basic PEI fingerprint, which indicates that the polymer backbone is fundamentally unchanged following the addition of ZnO and/or PVP. In the ZnO-containing membranes (PEI/ZnO-0.5, PEI/ZnO-1, PEI/PVP/ZnO-0.5, and PEI/PVP/ZnO-1) the imide carbonyl doublet at 1780/1720 cm^−1^ and the C–N band at 1360 cm^−1^ remain clearly visible. The preservation of these characteristic PEI bands is consistent with previous FTIR studies of PEI-based nanocomposites, where incorporation of inorganic fillers did not eliminate the characteristic imide absorptions [14,15]. In the low wavenumber region (500–700 cm^−1^) small changes in baseline intensity and slight broadening are observed; these bands overlap with the characteristic PEI ring-deformation region [14].

For PVP-containing membranes PEI-PVP, PEI/PVP/ZnO-0.5 and PEI/PVP/ZnO-1, the extra absorption peak about 1650 cm^−1^ is attributed to C=O stretching of the pyrrolidone ring of PVP [16,17]. This band is especially noticeable in the spectra of PEI/PVP/ZnO-1 (orange) and PEI/PVP/ZnO-0.5 (purple), where a shoulder appears between the imide peak at 1720 cm^−1^ and the aromatic band at 1600 cm^−1^. The presence of PVP suggests that some amount of PVP is still embedded in the membrane matrix after the phase-inversion procedure although PVP is a water-soluble polymer and mostly leached out during coagulation. The FTIR data reveal the successful development of the PEI-based membrane structure and additional components with no degradation of the polymer backbone.

### 3.2. Analysis of SEM

**Figure 2:**
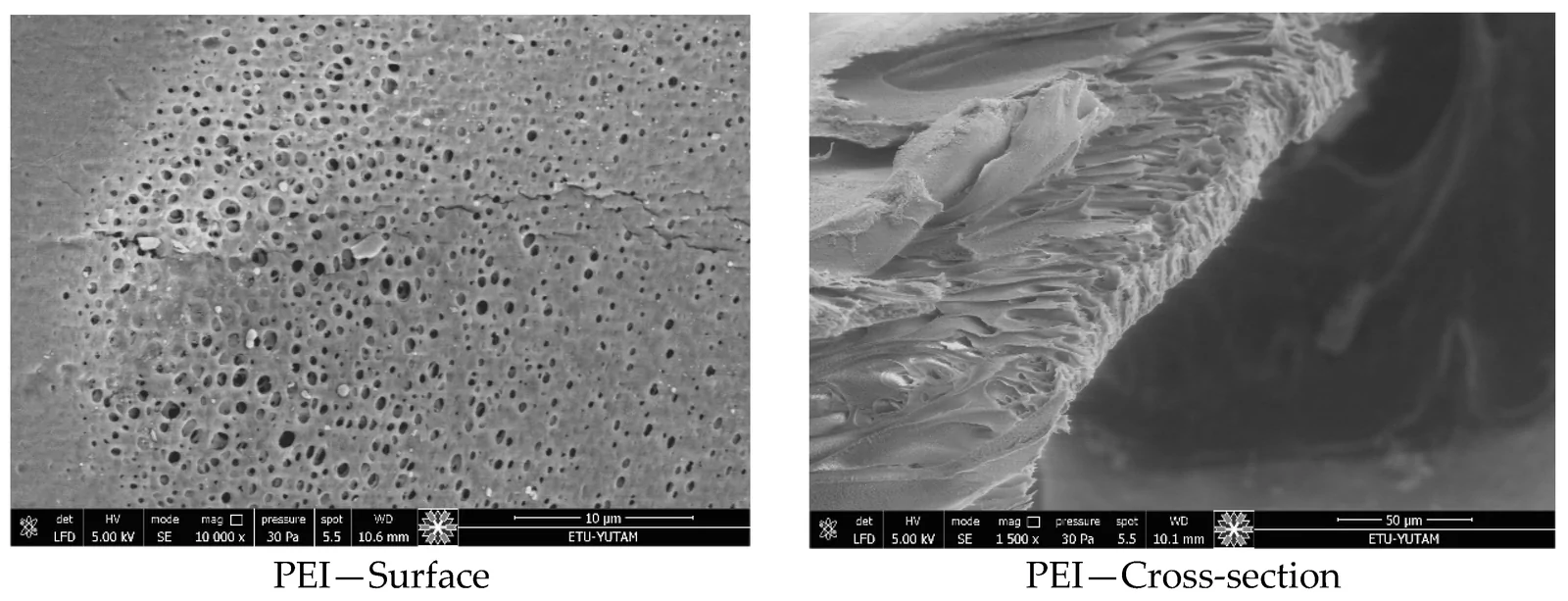

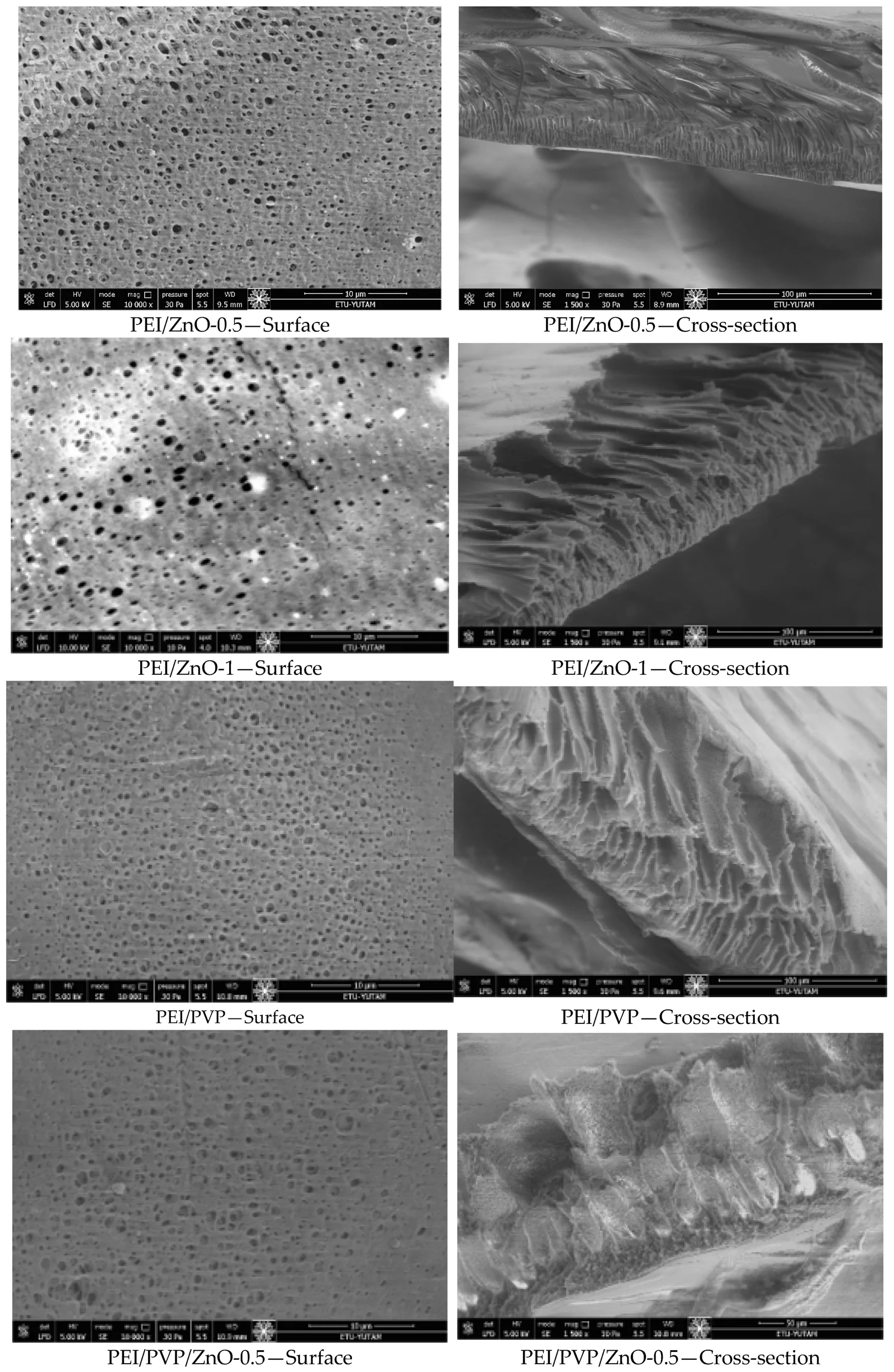

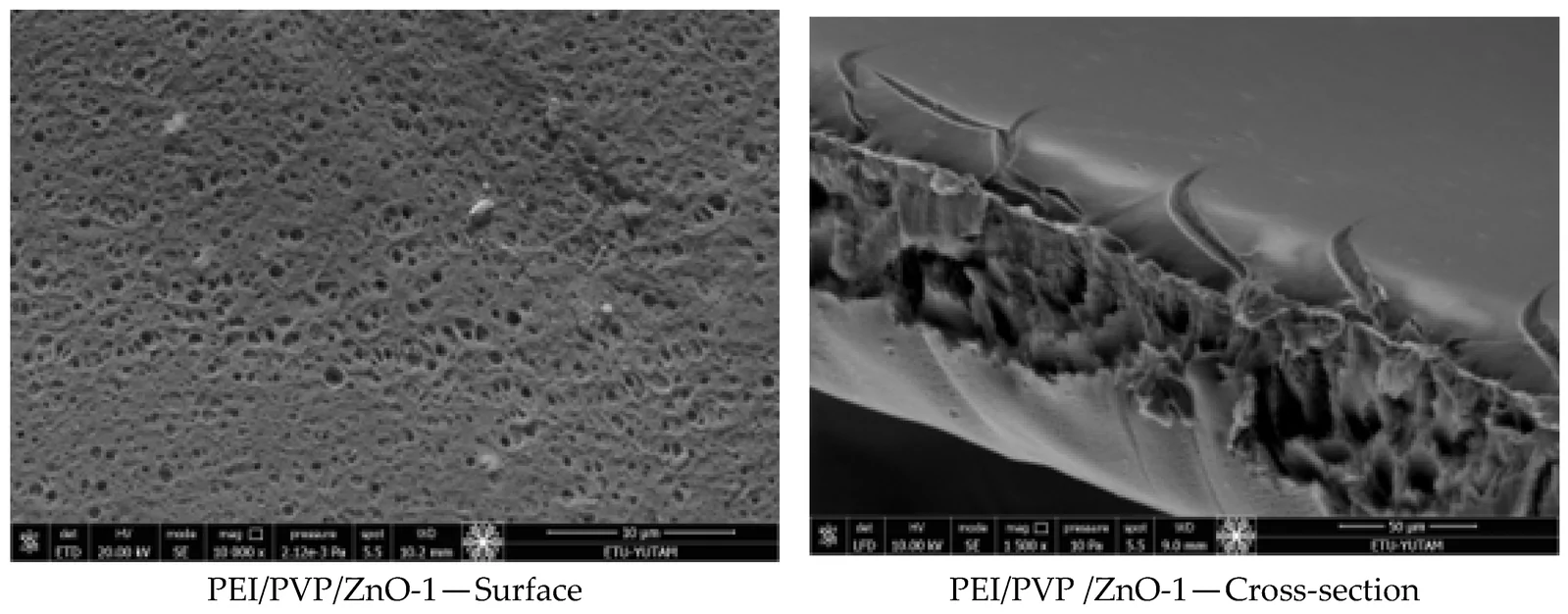
Surface and cross-sectional SEM micrographs of the fabricated membranes.

Figure 2 presents the SEM images of the surfaces and cross-sections of all fabricated membranes. Surface photos were taken at 10,000× magnification, and cross-sections were photographed at 1500×.

Plain PEI membrane. The surface micrograph (Figure 2, top-left) displays a modest density of small, approximately round pores dispersed over the membrane surface. The pore diameters are very uniform, but small, and certain areas of the surface appear heavier than others, with a minor surface break apparent on one side. The cross-section image shows the typical asymmetric structure that is expected from phase inversion: a dense skin layer on the top supported by a spongy sublayer. Similar asymmetric structures containing a dense selective layer and a porous substructure with finger-like macrovoids have been widely reported for phase-inversion membranes [16,17]. The relatively limited development of macrovoids in the plain PEI membrane may indicate a comparatively slower demixing process under the applied fabrication conditions.

PEI/ZnO-0.5 membrane. The surface morphology is similar to pristine PEI, except the pores seem slightly wider and more elongated, particularly in the higher parts of the micrograph. This suggests that even the little ZnO loading begins to influence the phase separation at the surface. The cross-section shows a very distinct structure with the formation of well-defined finger-like macrovoids extending from below the skin layer through a large fraction of the membrane thickness [18,19]. This demonstrates that 0.5 wt.% ZnO accelerates the solvent–non-solvent exchange rate which promotes immediate demixing and macrovoid formation.

PEI/ZnO-1 membrane. It is interesting to note that the surface of this membrane is much smoother and denser than the surfaces of both neat PEI and PEI/ZnO-0.5. The pores of the surface are more delicate and more rare. This could mean that a partial aggregation of nanoparticles near the surface at 1 wt.% loading results in a denser skin layer during the first moments of phase inversion. On the other hand, the cross-section reveals the most developed finger-like macrovoid structure of the ZnO-only series, with broad and regularly spaced channels penetrating deep into the body of the membrane. The paradox of increased density of surface and higher porosity of sublayer means that although the surface solidifies quickly due to higher ZnO content, the underlying structure is benefited with enhanced kinetics of phase separation.

PEI/PVP film. The presence of PVP as a pore former results in a much higher pore density on the surface of the membrane than in the neat PEI membrane. The pores are more equally distributed and slightly bigger, which is consistent with the hydrophilic PVP that allows water to enter faster during the phase inversion and then leaches out, leaving behind more pore channels. The cross-section shows well developed finger-like macrovoids that run across much of the membrane body with a transition to a spongy structure at the bottom [20,21]. The overall structure is more open than that of the neat PEI membrane, which suggests the efficiency of PVP as a pore former.

PEI/PVP/ZnO-0.5 membrane. The surface shows a good porosity with a dispersion of pores of different sizes similar to the PEI/PVP membrane. The cross-section, however, reveals a dramatically more open structure: the finger-like macrovoids are substantially larger and more prominent than in any of the previous membranes, forming large, teardrop-shaped channels that extend through much of the membrane thickness. At the bottom there is a spongy microporous layer. The combination of PVP and ZnO seems to exert a synergistic effect on the formation of macrovoids which is in agreement with the observation that both additives individually enhance the instantaneous demixing.

PEI/PVP/ZnO-1 membrane. This formulation gave the highest porosity in the surface of all the membranes. Surface microscopy indicates a very high density of well-dispersed holes. The cross-section shows very large interconnected macrovoid cavities with thin walls, the most open sublayer structure in this study. This morphology is the result of the synergy between PVP and the increased ZnO loading (1 wt.%), which maximizes the thermodynamic instability of the casting solution and results in the fastest phase separation [18,20,21].

### 3.3. The Viscosity

**Figure 3:**
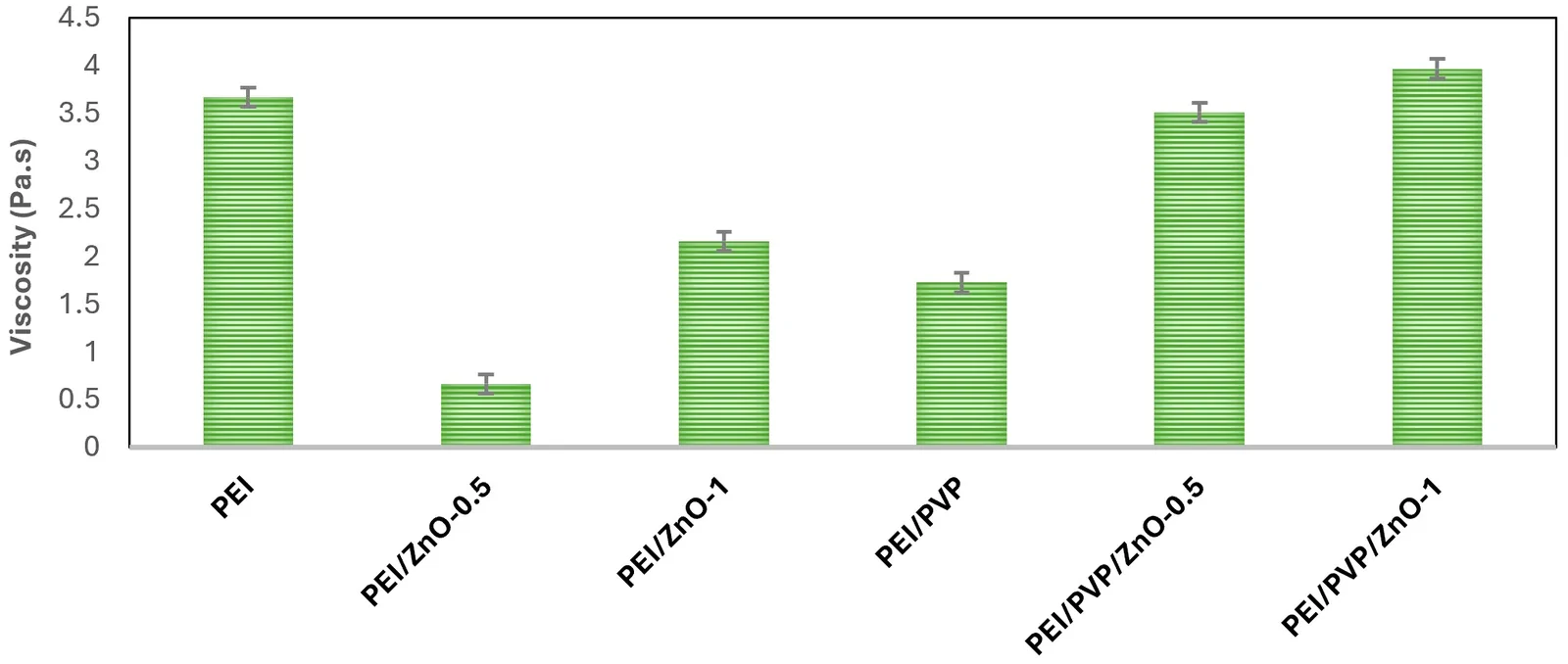
Viscosity of membrane casting solutions at 25 °C. (*SD* = 0.1, *n* = 3).

Viscosities of the membrane casting solutions are given in Figure 3. The viscosity of the neat PEI solution was about 3.65 Pa·s. A remarkable observation was that the addition of 0.5 wt.% ZnO without PVP significantly reduced the viscosity to about 0.7 Pa·s, which was the lowest value reported among all formulations. The observed behavior is due to the modest amount of well-dispersed ZnO nanoparticles disrupting the polymer chain entanglements, and lowering the intermolecular interactions in the PEI/NMP system. When the ZnO loading was increased to 1 wt.% (PEI/ZnO-1), the viscosity increased to about 2.15 Pa·s, indicating that at higher nanoparticle concentrations, the solid content and inter-particle interactions become sufficiently high to partially regain the viscosity, but still lower than that of the neat PEI solution. Similar concentration-dependent changes in the rheological behavior of polymer/nanoparticle casting solutions have been reported for nanocomposite membrane systems.

The viscosity of the PEI/PVP solution was around 1.75 Pa.s which was lower than that of the plain PEI. This reduction is expected as PVP is used to replace a fraction of the NMP solvent in the formulation (Table 1), and while PVP is a polymer itself, the net result at 7 wt.% loading is a reduction in total solution viscosity compared to the 15 wt.% PEI reference. In the presence of PVP, the viscosity trend showed a distinct difference compared to the PVP-free series: PEI/PVP/ZnO-0.5 had a viscosity of around 3.5 Pa·s and PEI/PVP/ZnO-1 attained the highest value of about 3.95 Pa·s. This indicates that the ZnO nanoparticles interact with PEI chains in addition to PVP in the presence of PVP, leading to enhanced polymer–nanoparticle networks that increase the solution viscosity. The differences in viscosity are important because they directly affect the kinetics of phase inversion. In particular, Han and Nam reported that PVP addition to PSF/NMP casting solutions increased the solution viscosity and introduced a kinetic resistance to phase separation, while its hydrophilic character simultaneously altered the thermodynamic driving force for demixing [17,22]. Lower viscosity solutions normally show faster solvent–non-solvent exchange and faster demixing, which favors macrovoid formation, as can be seen in the SEM cross-sections (Section 3.2) [23].

### 3.4. Measurements of Contact Angle

**Figure 4:**
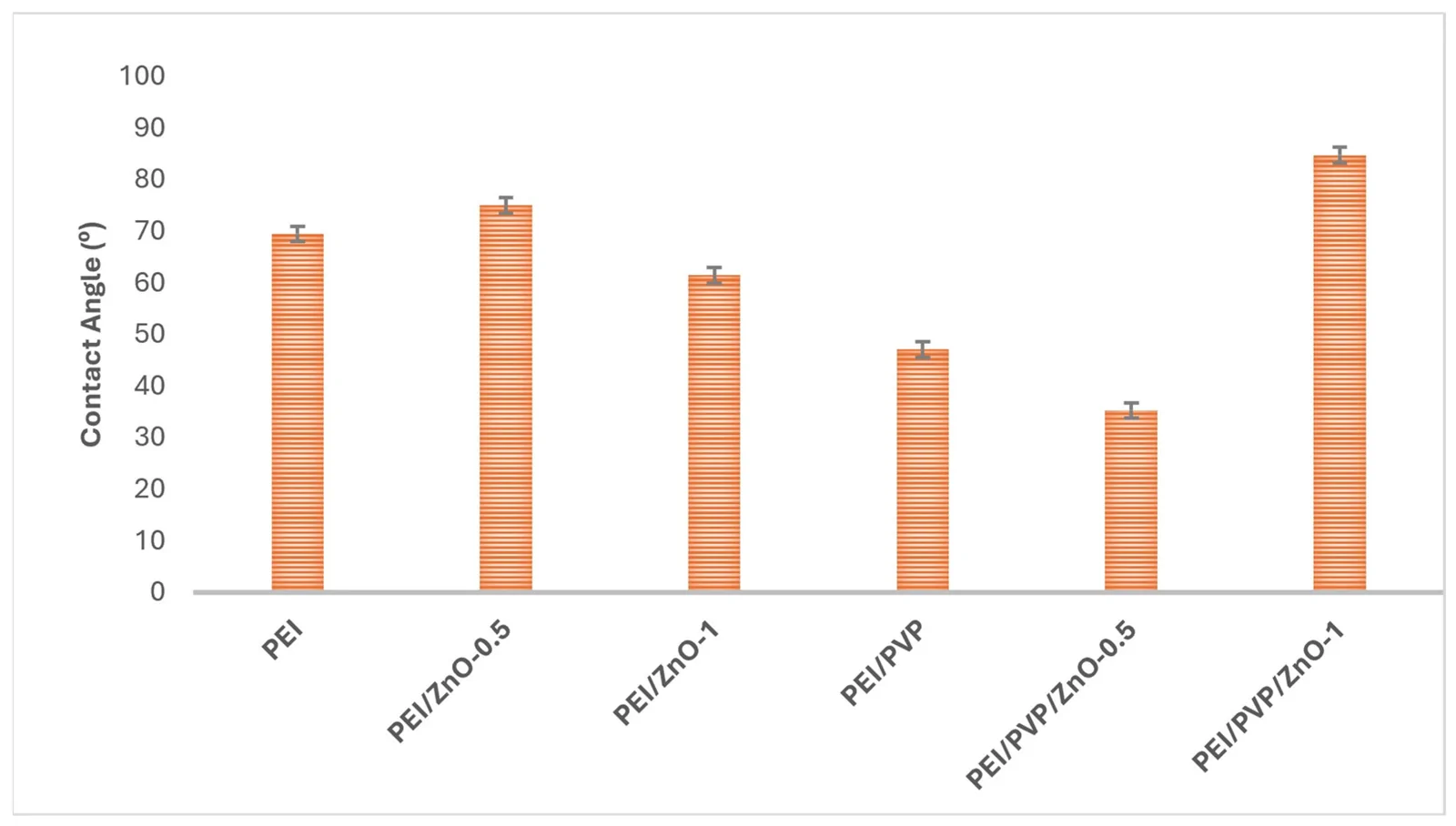
Water contact angle measurements of the fabricated membranes. (Data are presented as mean ± standard deviation (*SD* = 1.5, *n* = 3). Error bars represent *SD*.)

Figure 4 shows the water contact angle of the fabricated membranes. The plain PEI membrane showed a contact angle of ~70°, which is consistent with the moderately hydrophobic characteristic of polyetherimide. The ZnO-only series revealed that the PEI/ZnO-0.5 membrane had a slightly greater contact angle of ~75°, while the PEI/ZnO-1 membrane showed a lower value of ~62°. The initial increase at 0.5 wt.% ZnO could be attributed to the denser and smoother surface morphology observed in the SEM investigation (Section 3.2), since surface roughness and topography can significantly influence the apparent wetting behavior of polymeric surfaces [24,25]. At 1 wt.% ZnO, this effect appears to be counterbalanced by the intrinsic hydrophilic character of ZnO nanoparticles, resulting in a net increase in surface wettability [8,26].

The greatest reduction in contact angle was recorded for the PEI/PVP-containing membranes. The contact angle of the PEI/PVP membrane was about 47°, while the PEI/PVP/ZnO-0.5 formulation showed the lowest contact angle of about 35°, which was 50% lower than that of clean PEI. This significant improvement in wettability can be attributed to the combined contribution of residual hydrophilic PVP, confirmed by FTIR analysis (Section 3.1), and hydrophilic ZnO nanoparticles at the membrane surface [8,26]. PVP contains polar lactam carbonyl groups that can interact strongly with water, while ZnO nanoparticles possess hydrophilic surface sites that enhance their affinity toward water [8,26].

It is interesting to note that the contact angle of the PEI/PVP/ZnO-1 membrane was considerably greater, ~84°, which was the greatest among all the formulations. This surprising result could be associated with changes in surface morphology and possible nanoparticle aggregation at the higher ZnO loading. If aggregation and increased surface heterogeneity occur, the resulting surface topography may promote partial air entrapment beneath the water droplet and increase the apparent contact angle according to the Cassie–Baxter wetting model [27,28]. Overall, the trend confirms that the combination of PVP and moderate ZnO loading (0.5 wt.%) provided the most favorable hydrophilic surface among the investigated formulations, as evidenced by the lowest measured contact angle of approximately 35°.

### 3.5. Water Retention Capacity and Porosity

**Figure 5:**
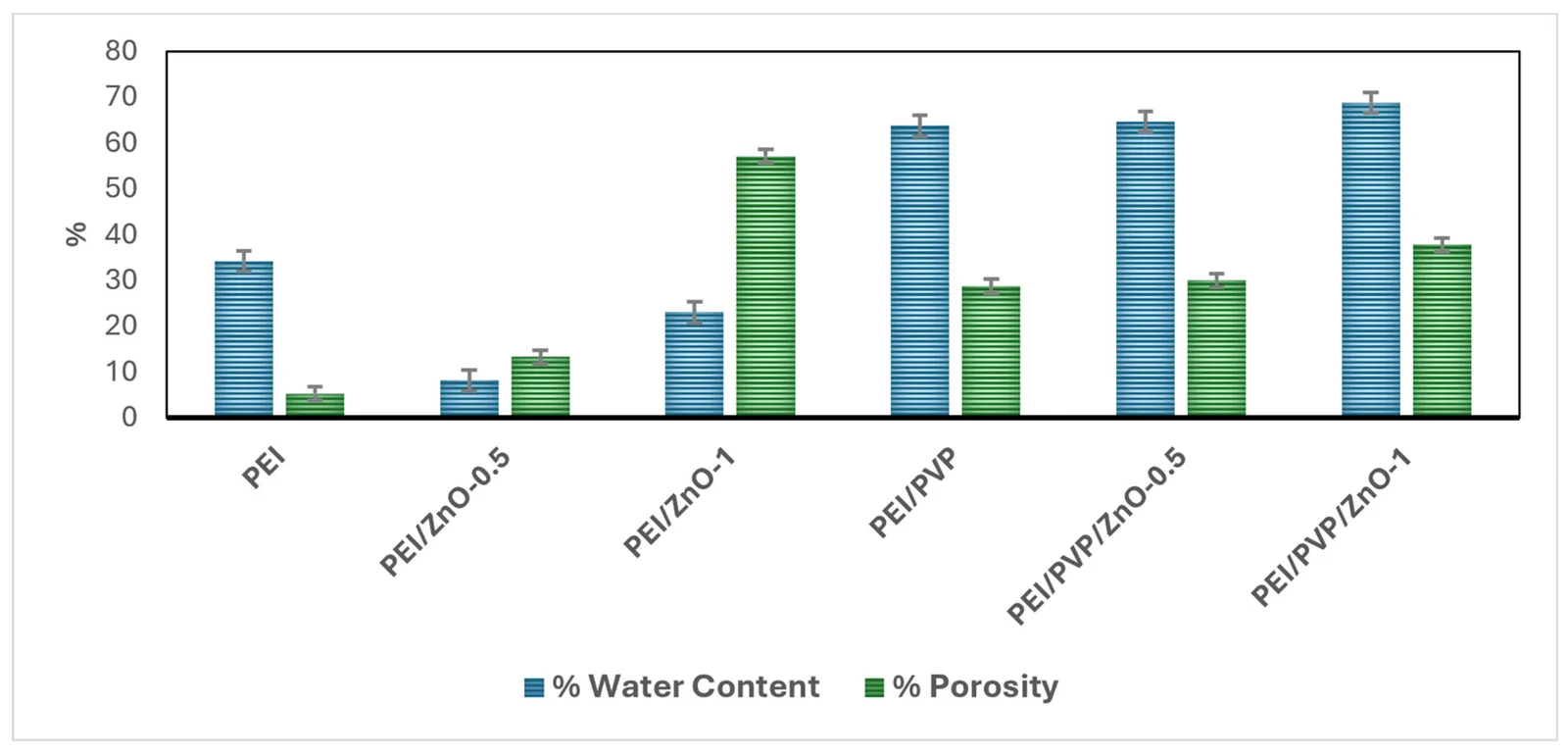
The water retention capacity and porosity of the fabricated membranes (*n* = 3, *For retention capacity SD* = 2.28, *n* = 3 *For porosity SD* = 1.5).

Figure 5 illustrates the water content and porosity values of the manufactured membranes. The neat PEI membrane exhibited a water content of approximately 34% and the lowest porosity of approximately 5%. The incorporation of ZnO nanoparticles without PVP resulted in a non-monotonic change in water content, whereas porosity increased markedly with increasing ZnO concentration. The PEI/ZnO-0.5 membrane exhibited a water content of approximately 8% and a porosity of approximately 13%, while the corresponding values increased to approximately 23% and 56%, respectively, for PEI/ZnO-1. The pronounced increase in porosity at 1 wt.% ZnO may be associated with changes in phase-inversion kinetics and membrane pore structure induced by nanoparticle incorporation [7,29]. However, the decrease in water content at 0.5 wt.% ZnO indicates that water retention was not governed solely by membrane porosity or the hydrophilic character of ZnO, and may also depend on pore connectivity, pore-size distribution, and the interactions between ZnO and the PEI matrix [7,29].

The PVP-containing membranes showed water content values of approximately 63–69% and porosity values of approximately 29–38%, which were substantially higher than those of the neat PEI membrane. The PEI/PVP membrane exhibited a water content of approximately 63% and a porosity of 29%, confirming the significant effect of PVP on water retention and membrane pore formation. The higher water content and porosity can be attributed to the hydrophilic character of PVP and its role as a pore-forming additive during phase inversion [21,24,30]. PVP is hydrophilic and its incorporation into polymeric casting solutions has been reported to increase equilibrium water content and modify membrane porosity [24,30]. Jasiewicz and Pietrzak, for example, demonstrated that PVP-modified PES membranes prepared by phase inversion exhibited increased equilibrium water content and substantial changes in porosity compared with the unmodified membrane [30]. Similarly, PVP-containing phase-inversion membranes have been reported to exhibit increased water content and porosity as a consequence of the hydrophilic character of PVP and its contribution to solvent–non-solvent mass transfer during membrane formation [24]. The addition of ZnO to the PVP-containing formulations resulted in a gradual increase in both water content and porosity. The PEI/PVP/ZnO-0.5 membrane exhibited a water content of approximately 64% and a porosity of approximately 30%, whereas these values increased to approximately 69% and 38%, respectively, for PEI/PVP/ZnO-1. Thus, in contrast to the previous interpretation, ZnO incorporation did not produce negligible changes in the PVP-containing membranes; rather, the higher ZnO loading promoted a moderate increase in both water retention and porosity. This behavior may be attributed to the combined effects of the hydrophilic character of ZnO and PVP, changes in phase-separation kinetics, and modifications in the membrane pore structure during phase inversion [7,29]. The relatively modest increase in porosity compared with the pronounced effect observed for PEI/ZnO-1 also suggests that the presence of PVP may modify the influence of ZnO on membrane formation and pore development. This observation is consistent with recent studies showing that the effect of ZnO on membrane porosity can be non-monotonic because nanoparticle addition simultaneously affects hydrophilicity, phase-separation kinetics, casting-solution viscosity, and possible pore obstruction [7,29].

### 3.6. Analysis of Average Pore Size

**Figure 6:**
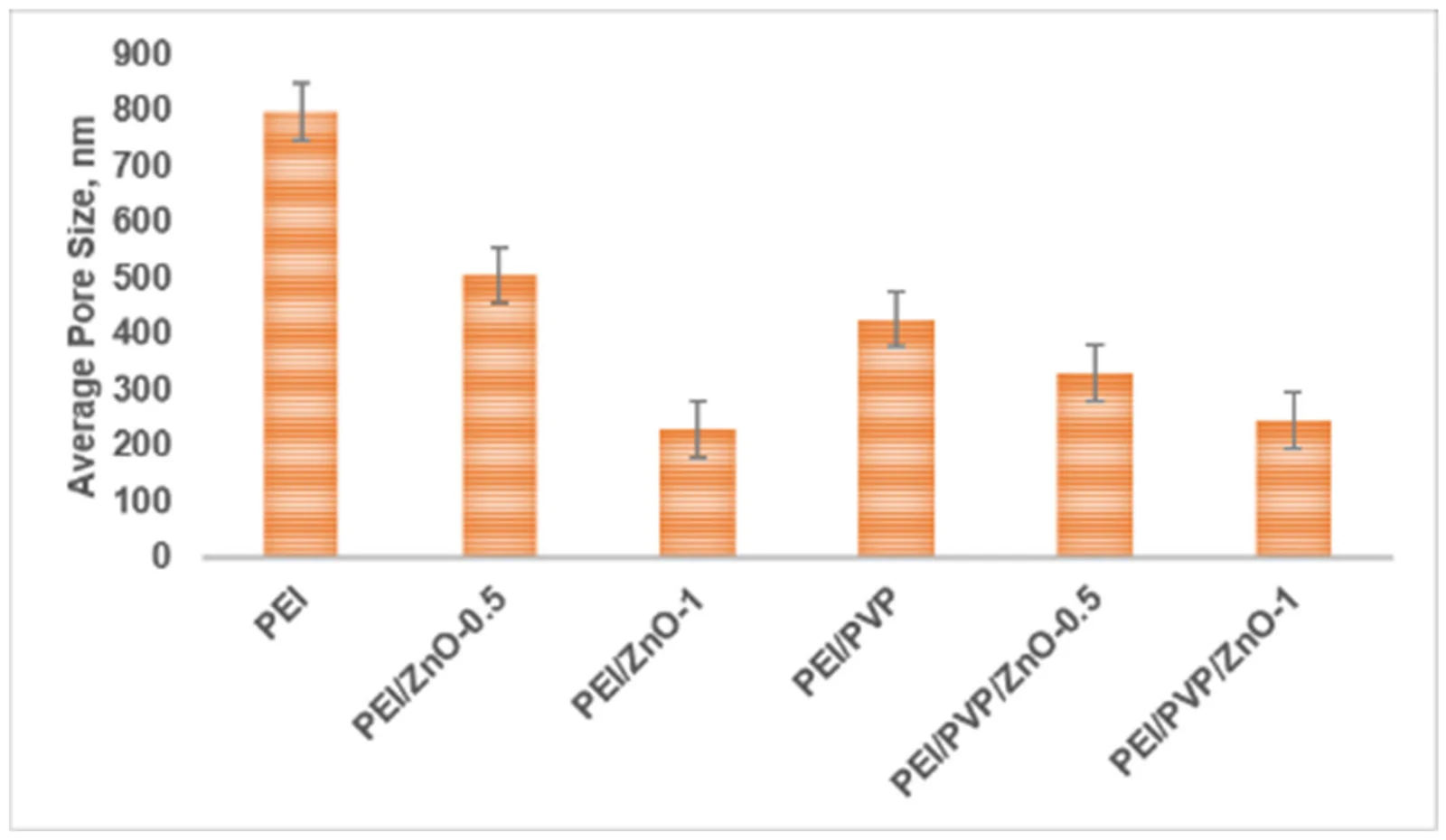
The mean pore size of the fabricated membranes (*SD* = 50, *n* = 3).

The mean pore diameters of the fabricated membranes are determined by the Guerout–Elford–Ferry equation and shown in Figure 6. This approach estimates the mean pore radius from experimentally determined membrane porosity, pure-water permeation rate, membrane thickness, effective membrane area, water viscosity, and applied pressure, and has been widely used for estimating the pore size of porous ultrafiltration membranes [25,31,32]. The clean PEI membrane showed the highest predicted mean pore radius, about 870 nm. This relatively high value, together with the modest surface pore density found in the SEM images (Section 3.2), suggests that the neat PEI membrane contains comparatively fewer but wider effective water-transport pathways. It should be noted, however, that the GEF equation provides an effective mean pore radius rather than a direct measurement of individual surface pores.

The computed average pore size decreased significantly with the incorporation of ZnO nanoparticles. The average pore size of the PEI/ZnO-0.5 membrane was found to be approximately 500 nm, decreasing further to approximately 220 nm for PEI/ZnO-1. Such changes in the calculated effective pore radius can arise from the combined effects of nanoparticle incorporation on phase-separation kinetics, surface-layer formation, membrane porosity, and water permeability [7,26]. The significant reduction may be associated with the formation of a denser selective surface layer in the presence of ZnO, as suggested by the SEM observations (Section 3.2), together with a redistribution of the pore structure toward finer effective transport pathways. The pronounced decrease from approximately 500 to 220 nm with increasing ZnO loading suggests that the higher nanoparticle concentration may promote greater structural densification and/or reduce the effective dimensions of the water-transport pathways. Similar changes in effective pore size associated with nanoparticle incorporation have been reported for nanoparticle-modified phase-inversion membranes [7,26].

The pore size of the membranes containing PVP was approximately 420 nm for PEI/PVP, 330 nm for PEI/PVP/ZnO-0.5, and 240 nm for PEI/PVP/ZnO-1, indicating a progressive decrease in effective pore size with increasing ZnO loading. The relatively large effective pore sizes obtained for the PVP-containing membranes are consistent with the known pore-forming effect of PVP during non-solvent-induced phase separation. PVP can modify solvent–non-solvent exchange and phase-separation kinetics, while its partial leaching during coagulation can contribute to the formation of additional pore channels [21,33]. The decreasing pore size from PEI/PVP to PEI/PVP/ZnO-1 suggests that ZnO incorporation progressively modifies the membrane morphology and reduces the effective dimensions of the transport pathways, despite the presence of macrovoids observed in the SEM cross-sections. Therefore, the GEF-derived pore radius should be interpreted as an effective hydraulic pore parameter rather than as a direct measurement of the physical diameter of individual pores observed by SEM [25,31,32].

### 3.7. Flux Performance of Distilled Water and Lake Water

**Figure 7:**
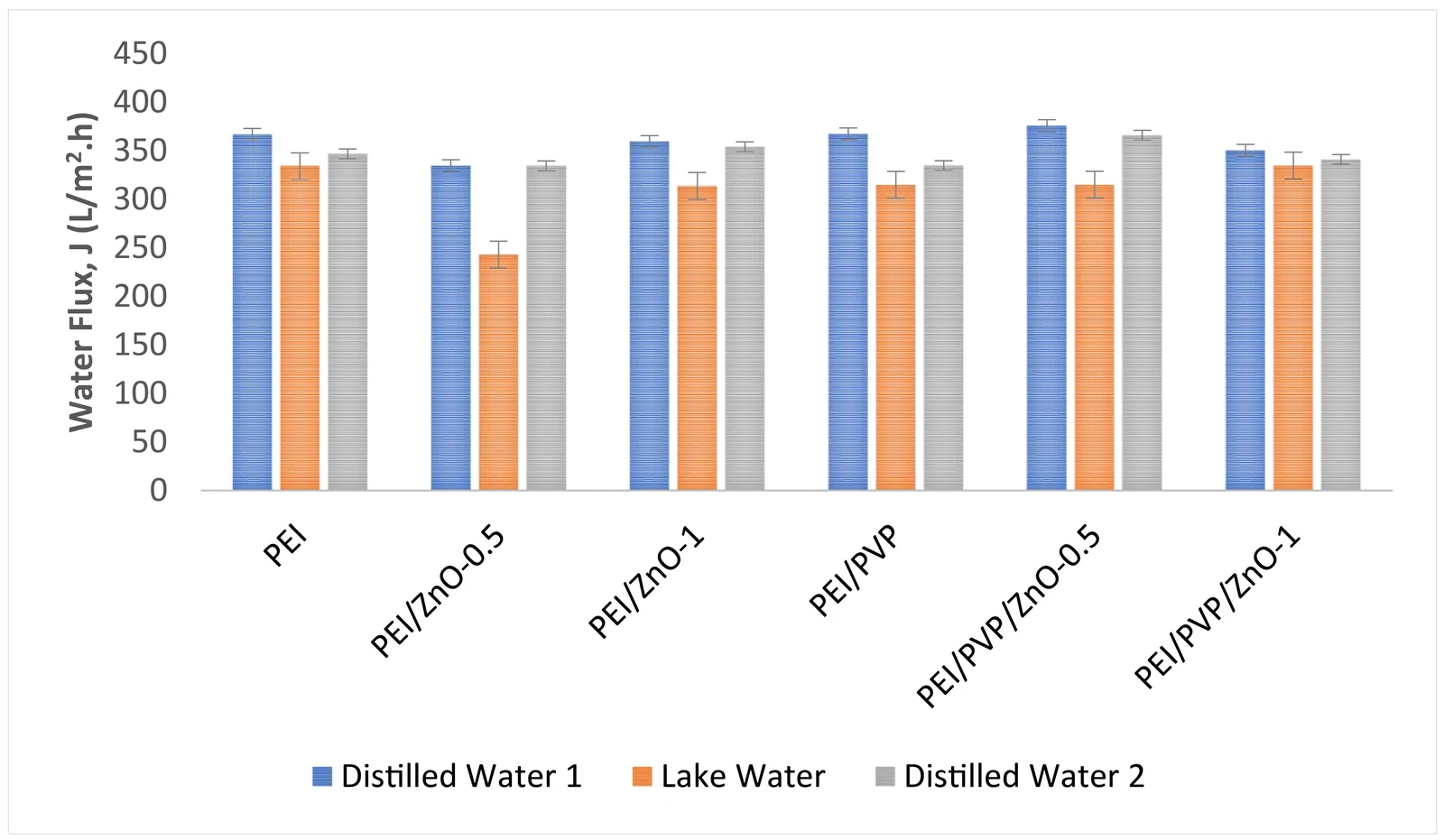
The distilled-water and lake-water flux performance of the fabricated membranes. (*SD* = 5, *n* = 3).

Figure 7 shows the flux performance of the fabricated membranes during the sequential filtration of distilled water, lake water, and distilled water. This three-step protocol allows for the determination of the initial permeation capacity and the flux recovery after exposure to a real foulant source. Sequential pure-water/foulant-water/pure-water filtration is commonly used to evaluate membrane fouling behavior by comparing the initial pure-water flux with the flux during foulant filtration and the subsequent recovered pure-water flux [34,35]. The flux recovery ratio (FRR) obtained after cleaning provides an indication of the fraction of fouling that is reversible rather than permanently associated with the membrane surface or pores [34].

The first pure-water fluxes of all membranes (Distilled Water 1) were pretty close to each other in the 315 to 370 L/m^2^·h range. The PEI/PVP/ZnO-0.5 and neat PEI membranes presented the highest initial fluxes of around 365–370 L/m^2^·h, followed by PEI/ZnO-1 (∼355 L/m^2^·h). The low initial flow of the PEI/PVP membrane (~315 L/m^2^·h) is in agreement with the smaller predicted pore size (Section 3.6) compared to the neat PEI membrane, despite the larger porosity. This observation supports the importance of the selective surface-layer characteristics and effective hydraulic pore size in determining water permeation, rather than bulk porosity alone. Similar behavior has been reported for PEI/PVP ultrafiltration membranes, where PVP incorporation modified the membrane pore structure, water permeability, and surface hydrophilicity [34].

All membranes showed a decrease in flux when switched to lake water due to fouling. The reduction in flux during the filtration of natural water is expected because lake water contains suspended particles, natural organic matter, colloidal species, and other foulants that can accumulate on the membrane surface and/or enter and block membrane pores [35]. PEI/ZnO-0.5 showed the most drastic reduction in flux, dropping to about 240 L/m^2^·h, a 29% decrease from the original value. This is remarkable as PEI/ZnO-0.5 has the smallest mean pore size (~50 nm) and may therefore be more susceptible to pore blockage by natural organic and colloidal foulants. However, the observed flux decline should also be considered in relation to surface hydrophilicity, pore connectivity, and membrane–foulant interactions rather than pore size alone [35]. On the other hand, the PEI/PVP/ZnO-1 membrane exhibited the most steady lake-water flux of about 330 L/m^2^·h, which was only a 3% decrease from the initial value of around 340 L/m^2^·h. The relatively stable flux of this membrane suggests a lower tendency toward irreversible foulant deposition under the present experimental conditions. Similar improvements in flux stability and fouling resistance have been reported for hydrophilic polymeric membranes modified with PVP and ZnO [26,34]. The clean PEI, PEI/ZnO-1, PEI/PVP and PEI/PVP/ZnO-0.5 showed substantial flux decline in the lake-water filtration (about 310–335 L/m^2^·h).

Following cleaning and rehydration in distilled water (Distilled Water 2), all membranes exhibited significant flux recovery, with final values in the range 330–350 L/m^2^·h. The PEI/ZnO-0.5 membrane showed a remarkable recovery from its low lake-water flux (~240 L/m^2^·h) to about 335 L/m^2^·h, indicating that a large fraction of the accumulated foulants could be removed by the applied cleaning procedure and that the observed fouling was predominantly reversible. ZnO-modified polymeric membranes have also been reported to exhibit improved flux recovery after fouling, which has been attributed to increased hydrophilicity and weaker foulant adhesion at the membrane surface [26]. The overall consistency of the Distilled Water 2 fluxes for all the formulations suggests strong structural stability of all the membranes and largely removable fouling during lake-water filtering.

### 3.8. Fouling Behavior

**Figure 8:**
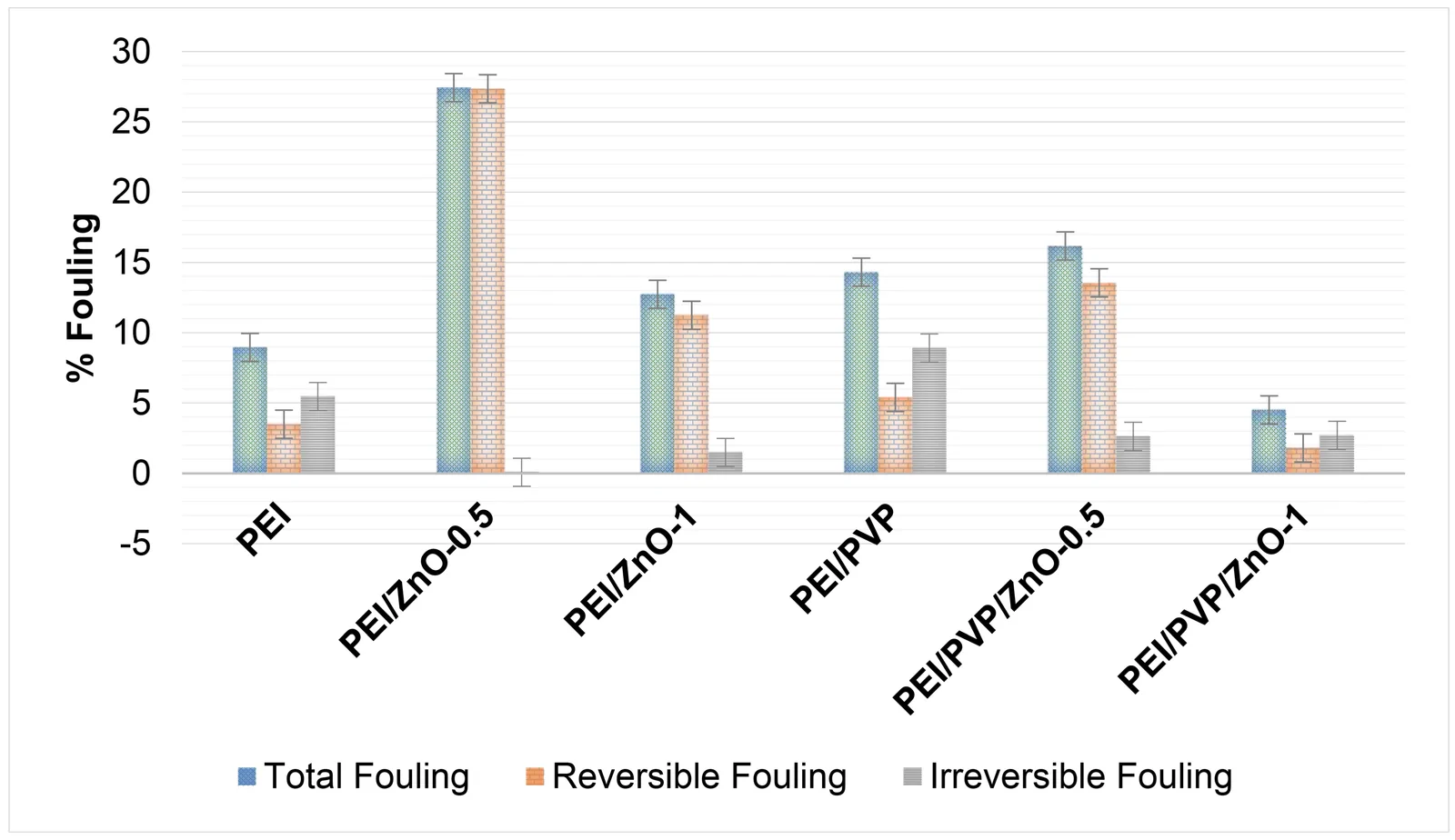
The fouling performance of the fabricated membranes: total, reversible, and irreversible fouling ratios.

Figure 8 shows the fouling performance of the membranes in terms of total fouling ratio, reversible fouling ratio and irreversible fouling ratio. This distinction is important because reversible fouling generally corresponds to loosely deposited foulants that can be removed by hydraulic or physical cleaning, whereas irreversible fouling is more strongly associated with adsorption and pore blocking and is more difficult to remove [36]. The neat PEI membrane exhibited a total fouling ratio of roughly 9.5% with a reversible fouling of about 3% and an irreversible fouling of about 5.5%. The moderate ratio of the irreversible fouling to the total fouling (about 58%) can reasonably be associated with the relatively hydrophobic character of unmodified PEI, which can promote hydrophobic interactions and adsorption of organic foulants [34].

Consequently, the PEI/ZnO-0.5 membrane showed the highest total fouling ratio (~28%), which was in accordance with the severe flux drop during lake-water filtering (Section 3.7). However, a key result was that most of this fouling was reversible (~27%) and only around 1% was permanent. This behavior indicates that although foulants accumulated substantially during lake-water filtration, most of the deposited material could be removed during the subsequent cleaning step. Similar behavior has been reported for ZnO-modified polymeric membranes, in which ZnO incorporation increased the fraction of reversible fouling and improved flux recovery [26,37]. The small pore size of PEI/ZnO-0.5 may contribute to pore blockage by colloidal and natural organic foulants; however, this effect should be considered together with surface hydrophilicity, surface charge, and foulant–membrane interactions [26,37]. The PEI/ZnO-1 membrane exhibited a reduced total fouling by ~13% with ~11% reversible and ~2% irreversible, confirming that the increased ZnO loading improves the fouling resistance while maintaining the predominantly reversible nature of fouling.

The total fouling ratio of the PEI/PVP membrane was around 14.5%, of which about 5% was reversible and about 9% was irreversible. The high irreversible fraction (∼62% of total) is not necessarily inconsistent with the hydrophilic character imparted by PVP, because fouling behavior depends not only on surface wettability but also on pore structure, foulant transport into the membrane, and specific foulant–polymer interactions [34,36]. The wider pore channels it forms (Section 3.6) may facilitate the penetration of some foulants into the porous substructure, potentially making a fraction of the deposited material more difficult to remove. This interpretation should be regarded as a possible mechanism rather than a definitive explanation.

The PEI/PVP/ZnO-0.5 membrane revealed a total fouling of ~16, with most being reversible (~14), with only ~2 being irreversible. The predominance of reversible fouling is consistent with the reported antifouling behavior of ZnO-containing polymeric membranes, where increased hydrophilicity and modified surface interactions can weaken foulant adhesion and facilitate cleaning [26]. PEI/PVP/ZnO-1 provided the best antifouling performance with the lowest total fouling ratio of about 4% which includes about 3% reversible and only about 1% irreversible fouling. This result indicates that the combined incorporation of PVP and ZnO substantially reduced the fraction of strongly attached foulants under the present experimental conditions. Similar synergistic improvements in fouling resistance have been reported for ZnO-containing polymeric membranes, although the optimum nanoparticle loading depends strongly on membrane composition and morphology [26,37]. The excellent result achieved here, considering the counterintuitively high contact angle of this membrane (Section 3.4), suggests that contact angle alone cannot fully explain the observed fouling behavior. Surface charge, pore architecture, nanoparticle distribution, and the nature of the foulants can also strongly influence foulant deposition and removal [26,37]. The possible contribution of surface roughness and heterogeneous nanoparticle distribution should therefore be considered as a hypothesis requiring further characterization, for example by AFM and surface chemical analysis, rather than as a confirmed mechanism. The fouling analysis overall shows that ZnO incorporation substantially altered the distribution between reversible and irreversible fouling in the present membrane series. The PEI/PVP/ZnO-1 composition showed the optimum combination of low total fouling and minimum irreversible fouling under the experimental conditions employed.

## 4. Discussion

The results show that the effects of ZnO and PVP on PEI membranes are strongly formulation-dependent rather than monotonic with ZnO loading. Across the six formulations, changes in casting-solution viscosity were accompanied by clear changes in surface and cross-sectional morphology. The PVP-free PEI/ZnO-0.5 solution had the lowest measured viscosity and developed pronounced finger-like macrovoids, whereas the PVP/ZnO formulations showed higher viscosities but also highly open cross-sectional structures. This combination indicates that the final morphology reflects both kinetic and thermodynamic effects during phase inversion; the present data do not support attributing the observed structures to viscosity alone.

FTIR confirmed retention of the PEI backbone and supported the presence of residual PVP, while the low-wavenumber feature associated with Zn–O remained tentative at the present loadings. The wetting response was likewise non-monotonic. PEI/PVP/ZnO-0.5 produced the lowest contact angle (approximately 35°), whereas PEI/PVP/ZnO-1 produced the highest (approximately 84°). Because PEI/PVP/ZnO-1 nevertheless exhibited the lowest total and irreversible fouling, contact angle alone cannot explain the fouling response. Surface roughness, pore architecture, nanoparticle distribution, and membrane–foulant interactions may all contribute, but additional surface characterization would be required to distinguish these effects.

The initial distilled-water fluxes occupied a comparatively narrow range (315–370 L/m^2^·h), whereas lake-water exposure differentiated the formulations more clearly. PEI/ZnO-0.5 underwent the largest flux decline but recovered strongly after cleaning, consistent with predominantly reversible fouling. PEI/PVP/ZnO-1 showed the smallest lake-water flux decline and the lowest total (approximately 4%) and irreversible (approximately 1%) fouling ratios under the present short-term protocol. These results indicate that the combined PVP/ZnO formulation can modify fouling behavior without imposing a large penalty on the initial water flux.

Two limitations should remain explicit. First, the gravimetric porosity values obtained for the ZnO-only membranes exceed the physical range of a void-volume fraction; those inputs and the GEF-derived pore-radius values therefore require verification before quantitative interpretation. Second, the present filtration experiments establish short-term behavior for one lake-water condition and do not demonstrate long-term structural stability or service life. Within these limits, the data show that ZnO alters the balance between reversible and irreversible fouling and that the PVP/ZnO combination produced the most favorable antifouling response among the formulations tested.

## 5. Conclusions

PEI-based membranes containing ZnO and/or PVP were fabricated by phase inversion and compared under common preparation and filtration conditions. The additives produced formulation-dependent changes in casting-solution viscosity, membrane morphology, surface wettability, water flux, and fouling behavior. PEI/PVP/ZnO-0.5 showed the lowest contact angle (approximately 35°), whereas PEI/PVP/ZnO-1 provided the most favorable short-term antifouling response, with the smallest lake-water flux decline and the lowest total (approximately 4%) and irreversible (approximately 1%) fouling ratios. The results also show that wettability alone does not predict fouling performance. Before quantitative use of the porosity and GEF-derived pore-radius results, the gravimetric porosity values for the ZnO-only membranes should be verified. Future work should examine a broader ZnO concentration range, independent membrane replicates, longer filtration/cleaning cycles, surface roughness and elemental distribution, and well-characterized wastewater matrices.

## Figures and Tables

**Table 1:** Composition and formulation of membrane casting solutions.

Membrane	PEI (wt.%)	PVP (wt.%)	NMP (wt.%)	ZnO (wt.%)
PEI	15	–	85	–
PEI-PVP	15	7	78	–
PEI-ZnO 0.5	15	–	84.5	0.5
PEI-ZnO 1	15	–	84	1
PEI-PVP-ZnO 0.5	15	7	77.5	0.5
PEI-PVP-ZnO 1	15	7	77	1

**Table 2:** Lake-water parameters.

Parameters	Concentrations
Alkalinity (mg/L)	153 ± 2.5
Chloride (mg/L)	62 ± 2
Conductivity (μs/cm)	448 ± 3
Dissolved Oxygen (mg/L)	8.43 ± 0.5
Fe (mg/L)	0.3 ± 0.01
Mn (mg/L)	1.2 ± 0.01
Nitrate (mg/L)	2.1 ± 0.2
pH	7.82 ± 0.03
Sulfate (mg/L)	32 ± 1
Total Organic Carbon -TOC (mg/L)	3.2 ± 0.3
Turbidity (NTU)	2.52 ± 0.03

## Data Availability

The original contributions presented in this study are included in the article. Further inquiries can be directed to the corresponding author.
